# European Consensus on Malabsorption—UEG & SIGE, LGA, SPG, SRGH, CGS, ESPCG, EAGEN, ESPEN, and ESPGHAN: Part 2: Screening, Special Populations, Nutritional Goals, Supportive Care, Primary Care Perspective

**DOI:** 10.1002/ueg2.70011

**Published:** 2025-03-15

**Authors:** Marco Vincenzo Lenti, Heinz Florian Hammer, Ilja Tacheci, Rosa Burgos, Stephane Schneider, Anastasiou Foteini, Aleksejs Derovs, Jutta Keller, Ilse Broekaert, Marianna Arvanitakis, Dan Lucian Dumitrascu, Oscar Segarra‐Cantón, Željko Krznarić, Juris Pokrotnieks, Gonçalo Nunes, Johann Hammer, Loris Pironi, Marc Sonyi, Cristina Maria Sabo, Juan Mendive, Adrien Nicolau, Jernej Dolinsek, Denisa Kyselova, Lucrezia Laterza, Antonio Gasbarrini, Teodora Surdea‐Blaga, Jorge Fonseca, Christos Lionis, Gino Roberto Corazza, Antonio Di Sabatino

**Affiliations:** ^1^ Department of Internal Medicine and Medical Therapeutics University of Pavia Pavia Italy; ^2^ First Department of Internal Medicine Fondazione IRCCS San Matteo Pavia Italy; ^3^ Division of Gastroenterology and Hepatology Department of Internal Medicine Medical University Graz Austria; ^4^ 2nd Department of Internal Medicine ‐ Gastroenterology University Hospital Hradec Králové Charles University Faculty of Medicine in Hradec Králové Hradec Kralove Czech Republic; ^5^ Endocrinology and Nutrition Department Hospital Universitari Vall d'Hebron Diabetes and Metabolism Research Unit Vall d'Hebron Institut de Recerca (VHIR) Universitat Autonoma de Barcelona Barcelona Spain; ^6^ Gastroenterology and Nutrition Centre Hospitalier Universitaire de Nice Université Côte d'Azur Nice France; ^7^ 4th Local Primary Care Team Municipality Practice and Academic Practice of Heraklion University of Crete Crete Greece; ^8^ Department of Internal Diseases Rīga Stradiņš University Riga Latvia; ^9^ Israelitic Hospital Academic Hospital University of Hamburg Hamburg Germany; ^10^ Department of Paediatrics Faculty of Medicine and University Hospital Cologne University of Cologne Cologne Germany; ^11^ Department of Gastroenterology Digestive Oncology and Hepatopancreatology HUB Hôpital Erasme Université Libre de Bruxelles Brussels Belgium; ^12^ 2nd Department of Internal Medicine Iuliu Hatieganu University of Medicine and Pharmacy Cluj‐Napoca Romania; ^13^ 2nd Medical Department Emergency Clinical County Hospital Cluj‐Napoca Romania; ^14^ Paediatric Gastroenterology and Clinical Nutrition Unit Vall d'Hebron Hospital Universitari Vall d'Hebron Barcelona Hospital Campus Barcelona Spain; ^15^ Department of Gastroenterology, Hepatology and Nutrition University of Zagreb Zagreb School of Medicine University Hospital Centre Zagreb Zagreb Croatia; ^16^ Centre of Gastroenterology Hepatology and Nutrition Pauls Stradiņš Clinical University Hospital Riga Latvia; ^17^ Gastroenterology Department Hospital Garcia de Orta Almada Portugal; ^18^ Egas Moniz Center for Interdisciplinary Research (CiiEM) Egas Moniz School of Health & Science Almada Portugal; ^19^ Department of Gastroenterology and Hepatology Medical University of Vienna Vienna Austria; ^20^ Department of Medical and Surgical Sciences University of Bologna Bologna Italy; ^21^ Centre for Chronic Intestinal Failure IRCCS Azienda Ospedaliero‐Universitaria di Bologna Bologna Italy; ^22^ Clinic for General Medicine, Gastroenterology, and Infectious Diseases Augustinerinnen Hospital Cologne Germany; ^23^ La Mina Primary Health Care Academic Centre Catalan Health Institute University of Barcelona Barcelona Spain; ^24^ Pediatric Gastroenterology Hepatology and Nutrition Unit Pediatric Department University Medical Center Maribor Maribor Slovenia; ^25^ Faculty of Medicine University of Maribor Maribor Slovenia; ^26^ Department of Hepatogastroenterology IKEM Prague Czech Republic; ^27^ Department of Translational Medicine and Surgery Università Cattolica del Sacro Cuore Rome Italy; ^28^ CEMAD Fondazione Policlinico Universitario A. Gemelli IRCCS Rome Italy; ^29^ Laboratory of Health and Society School of Medicine University of Crete Heraklion Greece

**Keywords:** breath test, coeliac disease, diarrhoea, enteropathy, nutrition, pancreatitis, weight loss

## Abstract

Malabsorption is a complex and multifaceted condition characterised by the defective passage of nutrients into the blood and lymphatic streams. Several congenital or acquired disorders may cause either selective or global malabsorption in both children and adults, such as cystic fibrosis, exocrine pancreatic insufficiency (EPI), coeliac disease (CD) and other enteropathies, lactase deficiency, small intestinal bacterial overgrowth (SIBO), autoimmune atrophic gastritis, Crohn's disease, and gastric or small bowel resections. Early recognition of malabsorption is key for tailoring a proper diagnostic work‐up for identifying the cause of malabsorption. Patient's medical and pharmacological history are essential for identifying risk factors. Several examinations like endoscopy with small intestinal biopsies, non‐invasive functional tests, and radiologic imaging are useful in diagnosing malabsorption. Due to its high prevalence, CD should always be looked for in case of malabsorption with no other obvious explanations and in high‐risk individuals. Nutritional support is key in management of patients with malabsorption; different options are available, including oral supplements, enteral or parenteral nutrition. In patients with short bowel syndrome, teduglutide proved effective in reducing the need for parenteral nutrition, thus improving the quality of life of these patients. Primary care physicians have a central role in early detection of malabsorption and should be involved into multidisciplinary teams for improving the overall management of these patients. In this European consensus, involving 10 scientific societies and several experts, we have dissected all the issues around malabsorption, including the definitions and diagnostic testing (Part 1), high‐risk categories and special populations, nutritional assessment and management, and primary care perspective (Part 2).

## 1 Introduction

In this second part of the consensus, we report the statements regarding the screening and special populations, the nutritional issues, and the primary care perspective. In Section 2, we specifically focus on special populations, including children and adolescents, pregnancy, and older individuals. The methodology has already been described in Part 1 of the consensus.

## 2 Screening and Special Populations

### 2.1 Which High‐Risk Groups Should be Screened for Malabsorption?

#### 2.1.1 Statement

Patients with high‐risk symptoms and conditions including iron deficiency anaemia, megaloblastic anaemia, chronic diarrhoea, type 1 diabetes mellitus, dermatitis herpetiformis, cryptogenic hypertransaminasemia, and short stature or growth retardation in children, premature or male osteoporosis, or unexplained infertility, should be screened for coeliac disease or other malabsorptive disorders. First‐degree relatives of coeliac disease patients should be screened as they have a high risk of having asymptomatic coeliac disease.

Coeliac disease (CD) manifests with varied symptoms, with notable differences across age groups. Key features include growth failure, weight loss, diarrhoea/steatorrhoea, and iron deficient anaemia (IDA), with low HDL levels significantly raising CD likelihood in IDA patients [1, 2, 3]. In children, common complaints are malabsorption, diarrhoea, and failure to thrive [4, 5, 6, 7], while adults frequently experience anaemia, abdominal pain, and hypertransaminasemia [8]. Endoscopy in anaemic patients reveals CD prevalence between 2.7% and 19.5%, and 8.5% in patients with diarrhoea [9, 10]. Approximately 10%–15% of patients with obscure IDA have CD [11, 12], and the prevalence in IDA patients with biopsy‐confirmed CD is 3.2% [13]. Other symptoms include mood changes, aphthous stomatitis, short stature, and delayed puberty [14, 15]. According to a systematic review and meta‐analysis, roughly 1 in 31 patients with IDA was found to have histologic evidence of CD [16].

Epidemiologic and clinical studies show increased CD prevalence among first‐degree relatives [17, 18, 19, 20], second‐degree relatives, and those with short stature or anaemia [19, 20]. Up to 23% of asymptomatic siblings have biopsy‐proven CD [21], with siblings showing the highest pooled prevalence (8.9%), followed by offspring (7.9%) and parents (3.0%). In infertility studies, CD prevalence is around 3.2% in cases of “unexplained infertility” [22]. Type I diabetes mellitus patients show a higher CD prevalence, with varied percentages across populations [23, 24, 25, 26].

CD is also prevalent in skin disorders like dermatitis herpetiformis (i.e., the dermatological manifestation of CD), alopecia, and in immunoglobulin A deficiency [27, 28, 29]. It is notably present in patients with osteoporosis, especially in pre‐menopausal females and males [30]. Finally, CD prevalence is increased in cryptic hypertransaminasemia [31] and autoimmune hepatitis [32, 33], with a prevalence in adults of 4%, and higher in children.

Pernicious anaemia, which is a form of megaloblastic anaemia, is one of the most common clinical manifestations of autoimmune gastritis, and may be the first clinical manifestation to occur, especially in adult/elderly patients. IDA is also very common in autoimmune gastritis, especially in younger patients [34, 35, 36, 37, 38, 39, 40]. Given the relatively high prevalence of autoimmune gastritis, patients with pernicious anaemia, or IDA, who have other autoimmune disorders, a first‐degree family history of autoimmune gastritis, infertility/miscarriage, dyspepsia, or neurological alterations should be screened for this condition [41, 42, 43, 44, 45]. Table 1 reports the main high‐risk conditions that should be screened for CD.

**TABLE 1:** Main disorders or conditions that warrant screening for coeliac disease (CD) due to their frequent association.

First‐degree relatives of CD patients
Autoimmune or immune‐mediated disorders Type 1 diabetes mellitus Autoimmune thyroid disease Addison's disease Autoimmune hepatitis Primary biliary cholangitis Primary sclerosing cholangitis Dermatitis herpetiformis Alopecia Sjögren's syndrome IgA nephropathy IgA deficiency Recurrent aphthous stomatitis
Gastrointestinal disorders Irritable bowel syndrome and functional diarrhoea Microscopic colitis Inflammatory bowel disease Autoimmune gastritis Hypertransaminasemia Idiopathic pancreatitis
Neuropsychiatric disorders Epilepsy with occipital calcifications Neuropathy Chronic fatigue syndrome Ataxia
Reproductive disorders Delayed menarche Premature menopause
Genetic syndromes Turner syndrome Down syndrome Williams syndrome
Endocrinological or metabolic disorders Growth retardation or short stature Unexplained osteoporosis Dental enamel hypoplasia
Haematological or oncological disorders Unexplained iron deficiency anaemia or other nutrient‐deficient anaemia Spleen atrophy or hypofunction

#### 2.1.2 Statement

Symptomatic patients with terminal ileum resection/right hemicolectomy, cholecystectomy, irritable bowel syndrome and functional diarrhoea should be tested for bile acid malabsorption. If testing is not available, a trial with bile acid sequestrants should be considered.

Diarrhoea can result from malabsorption of bile acids, associated with Crohn's disease, CD, right hemicolectomy, cholecystectomy, microscopic colitis, fibrosis following radiotherapy [46, 47, 48, 49]. An increased prevalence (38%) of bile acid diarrhoea has been reported in diarrhoea‐IBS and functional diarrhoea [47, 48, 49, 50]. In a study in patients undergoing SeHCAT scan for chronic diarrhoea, the odds ratio OR for bile acid malabsorption was 2.5 for cholecystectomy, 12.4 for Crohn's ileal resection/hemicolectomy, and 7.9 for other reason ileal resection/hemicolectomy. Among 77 IBS‐D patients 27.3% tested positive [48]. A meta‐analysis confirmed a pooled rate of 28.1% (22.6%–34%) for bile‐acid malabsorption in IBS‐D patients [51]. Finally, SIBO could also co‐occur with bile acid malabsorption in patients who underwent right hemicolectomy/terminal ileum resection, and therefore should be considered.

#### 2.1.3 Statement

Patients with gastrointestinal symptoms and type 1 or 2 diabetes mellitus, metabolic syndrome, HIV infection, primary pancreatic disorders, and alcohol abuse should be screened for exocrine pancreatic insufficiency.

Chronic diarrhoea, weight loss, and steatorrhoea may depend on the severity of EPI. EPI prevalence was high in type 1 or 2 diabetes mellitus, metabolic syndrome, HIV, and high alcohol intake. Screening for EPI should be recommended in these patients, especially with gastrointestinal complaints [52]. Steatorrhoea can also develop after pancreaticoduodenectomy [53]. Other causes of EPI that should be considered include chronic tabagism, pancreatic resection‐radiotherapy‐chemotherapy, pancreatic adenocarcinoma, chronic pancreatitis, intraductal papillary mucinous neoplasms (IPMN), acute necrotic‐emorrhagic or autoimmune pancreatitis, and bilio‐digestive derivative surgery.

#### 2.1.4 Statement

Patients with malabsorption symptoms occurring after certain drug exposures, especially olmesartan or other angiotensin‐II receptor blockers, angiotensin‐converting enzyme inhibitors, mycophenolate, and immune check‐point inhibitors should be investigated for sprue‐like enteropathy. Patients with gastrointestinal symptoms after pelvic radiotherapy may have malabsorption.

There are reports on drug‐induced sprue‐like enteropathy with several compounds, including chemotherapeutics, immunosuppressants, immunotherapy, and angiotensin‐II receptor blockers (ARBs). In one study that included 465443 patients, a sprue‐like enteropathy was present in a certain proportion of patients treated with olmesartan, other ARBs, and angiotensin‐converting enzyme inhibitors [54]. Overall, there were 23 cases of intestinal malabsorption, with the highest incidence occurring with ARBs. According to a very recent systematic review, olmesratan is responsible for most cases of sprue‐like enteropathy, while this condition should be considered exceptional with other ARBs [55]. Recently, some authors have also reported the development of villous atrophy after treatment with immune check‐point inhibitors [56, 57, 58]. This condition is emerging after the widespread use of immune checkpoint‐inhibitors.

Small bowel irradiation can result in diarrhoea or malabsorption in up to 30% of patients [59]. After pelvic radiotherapy, occasionally patients have symptoms/signs of malabsorption, due to functional short bowel, bacterial overgrowth, vitamin B12 deficiency, pancreatic insufficiency, fatty acid malabsorption, or bile salt malabsorption [60].

### 2.2 Are There Any Specific Issues for Special Populations (i.e., Childhood, Pregnancy, Elderly)?

Sustained malabsorption in the developing child and adolescent can have long‐lasting effects on appropriate height, head circumference, and the development of cognitive skills or immune function in severe cases [61]. Therefore, early identification and management of malabsorption causing undernutrition are critical. The goal in the treatment of malabsorption is to treat the underlying cause (e.g., in CD, IBD, chronic liver disease) [61, 62, 63, 64], to promote maximal adaptation of the remaining gut (e.g., in intestinal failure [IF]) [65] and to substitute and monitor for deficiencies in energy, macro‐ and micronutrients.

#### 2.2.1 Statement

In children and adolescents with clinical signs of malabsorption, initial testing for coeliac disease, consisting of anti‐tissue transglutaminase‐IgA and total serum IgA, is recommended. If anti‐tissue transglutaminase‐IgA levels are > 10 times the upper limit of normal, duodenal biopsies can be omitted.

CD is one of the most frequent immune‐mediated gastrointestinal diseases in children and adolescents causing malabsorption [66]. Due to its heterogeneous and even asymptomatic presentation, CD is still underdiagnosed in childhood and adolescence. On duodenal biopsies, characteristic changes are intraepithelial lymphocytosis, crypt hyperplasia and various degrees of villous height reduction with complete restitution upon restriction of gluten in the diet [67]. Classical symptoms of malabsorption include failure to thrive, weight loss, and chronic diarrhoea [61]. Children and adolescents with diarrhoea‐predominant IBS‐like symptoms, iron deficiency anaemia, chronic constipation, and enamel defects have an increased prevalence of CD [68, 69, 70]. For initial testing, the combination of transglutaminase‐IgA and total IgA is sufficient and the most accurate [62]. If transglutaminase‐IgA levels are more than 10 times the upper limit of normal in two lab samples along with positive endomysial‐IgA the diagnosis of CD can be made (no‐biopsy approach) [69]. In children and adolescents with elevated transglutaminase‐IgA, but lower titres (< 10 times upper limit of normal), duodenal biopsies should be obtained to eliminate the risk of a false positive diagnosis [69]. As in children and adolescents with type 1 diabetes transglutaminase‐IgA may be low or fluctuating without abnormalities on duodenal biopsies, serological diagnosis should be complemented by histology [71]. HLA‐testing and the presence of symptoms are no more mandatory for a serology‐based diagnosis without duodenal biopsies [62, 69].

#### 2.2.2 Statement

Genetic and non‐genetic causes of carbohydrate malabsorption can be quite common in childhood and adolescence, and children and adolescents may show symptoms after ingestion of the respective carbohydrate, depending on the ingested dose and concurrent diseases like irritable bowel syndrome. Dietary restriction of the specific carbohydrate(s) is recommended when the intolerance to the specific carbohydrate is proven by validated symptom assessment.

Intolerance to carbohydrates is relatively common in childhood and adolescence and the prevalence has increased during the last few decades due to increased carbohydrate consumption [72]. Development of symptoms after ingestion of carbohydrates may or may not be related to carbohydrate malabsorption [73]. Carbohydrate maldigestion is caused by a deficiency of digestive enzymes (like lactase or sucrose‐isomaltase) and malabsorption is caused by an overloading of a transport system on the brush border of the epithelium in the small intestine [1, 74]. Non‐absorbed carbohydrates in the intestinal lumen result in osmotic fluid shifts into the lumen [75, 76], and are fermented by gut microbiota to gas [77, 78]. The child or adolescent may present with abdominal pain, nausea, bloating, flatulence, increased gut motility, and diarrhoea [74]. Extraintestinal symptoms, such as headache, vertigo, memory impairment, and lethargy have been described in less than 20% of patients and may be the result of toxic metabolites produced by sugar fermentation of colonic bacteria [79].

Genetic causes of carbohydrate malabsorption are lactase deficiency, congenital sucrase‐isomaltase deficiency (CSID), and glucose‐galactose malabsorption [80, 81, 82]. In congenital lactase deficiency, a rare autosomal recessive disease, where enzymatic activity is absent or reduced from birth on, symptoms start upon ingestion of lactose containing mother's own milk or formula. In adult‐type lactase deficiency, which is the most frequent cause of carbohydrate malabsorption, there is a developmentally regulated decline of the lactase gene [83] which varies among ethnic groups. Secondary lactase deficiency is a transient condition due to intestinal damage secondary to for example, gastrointestinal infections, CD, Crohn's disease, or SIBO. Diagnosis of lactase deficiency is by genetic testing (in the case of congenital lactase deficiency), while of lactose malabsorption is by hydrogen breath testing, and of lactose intolerance is by validated symptom testing [1, 73, 84, 85]. The same tests are available for adults [86]. In secondary lactase deficiency, lactose restriction is only necessary for a limited time and if symptoms can be attributed to lactose intolerance by validated symptom assessment. Children or adolescents with lactose intolerance who are treated with lactose reduced diet are at risk for a lower calcium intake; therefore, calcium supplementation and calcium fortified foods are recommended [74, 80].

In CSID, a rare autosomal recessive disease, symptoms typically occur after weaning in infancy upon exposure to sucrose and starch. The diagnosis is established by genetic testing and treatment consists in dietary restriction and enzyme replacement with an oral solution containing sacrosidase (Sucraid) [82].

Glucose‐galactose malabsorption is a very rare autosomal recessive disorder caused by a defect of a Na+/glucose co‐transporter causing diarrhoea soon after birth. Genetic testing confirms the diagnosis, and treatment consists of a low concentration of glucose‐galactose in the diet and a fructose formula in early life [87].

Non‐genetic carbohydrate intolerances such as for example, fructose intolerance, which is common in childhood and adolescence [88, 89], and sorbitol intolerance are diagnosed by obtaining a dietary history and a validated symptom questionnaire before and after ingestion of the respective carbohydrate [73]. Treatment consists of a reduction of intake of the responsible carbohydrate, a balanced intake of fructose and glucose, or a low sorbitol diet, respectively [74]. For symptoms of fructose intolerance in some countries the enzyme D‐xylose isomerase is available as a food supplement [90], which converts fructose into glucose.

#### 2.2.3 Statement

In children and adolescents with clinical signs of malabsorption (especially fat malabsorption), it is recommended to assess exocrine pancreatic function with faecal elastase measurement. In case of exocrine pancreatic insufficiency, it is recommended to monitor fat‐soluble vitamin levels and screen for micronutrient deficiency regularly, and substitute if necessary.

EPI in children and adolescents can be caused by (1) insufficient pancreatic stimulation (e.g., in CD by reduced release of cholecystokinin from the duodenum), (2) reduced synthesis of pancreatic juices (e.g., by damaged pancreatic tissue in cystic fibrosis or chronic pancreatitis), (3) obstruction of the pancreatic duct (e.g., in cystic fibrosis or in anatomical defects of the pancreatic ducts) [91, 92] (Table 2).

**TABLE 2:** Causes of exocrine pancreatic insufficiency in childhood and adolescence.

Genetic causes	Non‐genetic causes
Cystic fibrosis	Idiopathic EPI
Schwachman‐Bodian‐diamond syndrome	Coeliac disease
Johanson‐Blizzard syndrome	Idiopathic pancreatitis (chronic or acute recurrent)
Pearson anaemia	Autoimmune pancreatitis
Hereditary pancreatitis (chronic or acute recurrent)	

Abbreviation: EPI, exocrine pancreatic insufficiency.

The diagnosis of EPI can be made directly by measurement of pancreatic enzymes in duodenal secretions after secretin stimulation [93, 94]. The advantages are the high sensitivity and specificity and the possibility to measure bicarbonate, but the disadvantage is that the test is invasive and only available in specialist centres. Indirect diagnostic tests are faecal elastase, the fat absorption coefficient and the ^13^C‐mixed triglyceride breath test. Measurement of faecal elastase is simple and non‐invasive, but it is not useful in monitoring pancreatic enzyme replacement therapy (PERT), and it may be false positive in watery stools or intestinal inflammation. The ^13^C‐mixed triglyceride breath test is a non‐invasive test which may be used to monitor response to PERT, but disadvantages are the prolonged testing time (6 h), costs and the very limited availability [93, 94].

Children and adolescents with EPI need individualized dietary counselling for a balanced diet and PERT. Co‐therapy with proton pump inhibitors may improve the absorption of bile acids and reduces the inactivation of pancreatic enzymes by gastric acid. More specifically, inhibition of gastric acid secretion may help in inhibiting the inactivation of pancreatic enzymes by low pH. Liposoluble vitamin supplementation should be administered when necessary.

#### 2.2.4 Statement

Intestinal failure in childhood and adolescence is a rare condition with heterogeneous clinical manifestations and can include significant fluid, electrolyte, and nutrient malabsorption requiring intravenous supplementation. Due to the complexity of the disease a multidisciplinary team approach is recommended.

IF is defined as a reduction of functional gut mass below the minimal amount necessary for digestion and absorption adequate to satisfy the nutrient and fluid requirements for growth in children and adolescents [95]. IF is a heterogeneous group of rare conditions including short‐bowel syndrome in *sensu stricto* (intestinal resection for acquired or congenital gastrointestinal diseases), disorders of gastrointestinal motility (e.g., paediatric intestinal pseudo‐obstruction) and congenital enterocyte disorders (e.g., microvillus inclusion disease, tufting enteropathy) [96, 97] (Table 3). IF is associated with excessive fluid loss, nutrient malabsorption, electrolyte abnormalities, increased susceptibility to infections, PN associated complications and affects weight gain and growth.

**TABLE 3:** Causes of intestinal failure in children and adolescents.

Surgical short‐bowel syndrome	Intestinal resection for congenital gastrointestinal diseases	Congenital intestinal anomalies (e.g. volvulus, intestinal atresia, gastroschisis)
	Intestinal resection for acquired gastrointestinal diseases	For example, necrotising enterocolitis, Crohn's disease
Disorders of gastrointestinal motility		For example, paediatric intestinal pseudo‐obstruction, long‐segment Hirschsprung's disease, extensive intestinal aganglionosis
Congenital enterocyte disorders	Epithelial electrolyte transport disorders	For example, congenital chloride diarrhoea, congenital sodium diarrhoea
	Epithelial nutrient transport disorders	For example, glucose‐galactose malabsorption
	Disorders of epithelial enzymes and metabolism	For example, congenital lactase deficiency, sucrase‐isomaltase deficiency, DGAT1 deficiency, Hennekam lymphangiectasia‐lymphoedema syndrome‐1, PLVAP deficiency, abetalipoproteinemia, dyskeratosis congenita
	Disorders of epithelial trafficking and polarity	For example, microvillus inclusion disease, tufting enteropathy, trichohepatoenteric syndrome, MIRAGE syndrome
	Disorders of enteroendocrine cell dysfunction	For example, Mitchell‐Riley syndrome
	Immune dysregulation‐associated enteropathy	For example, IPEX, CD55 deficiency, XIAP‐deficiency

Management of IF is aimed at supporting adequate nutrition and minimizing the risk of complications, with the goal of independence from PN [96]. The optimisation of administration of fluids, electrolytes and nutrients via the parenteral and enteral route is ideally achieved via a multidisciplinary team approach [98, 99]. Enteral nutrition should be started early after bowel surgery beginning with mother's own milk or amino‐acid‐based formulae [100]. Micronutrient (vitamin and mineral) supplementation is a critical aspect of nutritional therapy. The anatomical location of the bowel resection determines the incidence and severity of micronutrient deficiencies. The likelihood of adequate bowel adaptation depends on the extent of gastrointestinal resection and the remaining absorptive capacity and is higher in case of longer residual small bowel, younger age at the time of surgery, preservation of the ileocaecal valve, diagnosis of necrotizing enterocolitis, absence of severe liver disease, and normal gastrointestinal motility [96].

#### 2.2.5 Statement

In children and adolescents with inflammatory bowel disease (especially Crohn's disease) screening and prevention of malnutrition and micronutrient deficiencies, prevention of osteoporosis and promotion of optimal growth and development are strongly suggested.

Malabsorption and malnutrition can occur in both ulcerative colitis and Crohn's disease, although it is a greater problem in Crohn's disease which can affect any part of the gastrointestinal tract, unlike ulcerative colitis, which is restricted to the colon with few direct malabsorptive consequences. Children and adolescents with IBD need screening for and prevention of malnutrition and micronutrient deficiencies, prevention of osteoporosis, and promotion of optimal growth and development [101].

At time of diagnosis, malnutrition is common in Crohn's disease and may persist despite treatment [102, 103]. Growth failure is due to a combination of inflammation, chronic malnutrition and prolonged corticosteroid use. Corticosteroid treatment has shown to increase the net loss of protein in Crohn's disease whereas enteral nutrition decreases proteolysis and increases protein synthesis [104, 105]. Micronutrient status including iron stores, zinc, vitamin D, should be checked on a regular basis and supplemented when needed as deficiencies may cause anaemia, impaired growth and poor bone health [101]. In Crohn's disease patients who have significant terminal ileal disease or who have undergone terminal ileal resection, vitamin B12 should be monitored and substituted if needed [101]. In children and adolescents treated with methotrexate of sulfasalazine, folic acid should be supplemented [106]. In steroid‐treated IBD patients, an adequate supply of calcium and vitamin D should be ensured [107]. Exclusive enteral nutrition is effective and is recommended as the first line treatment to induce remission in children and adolescents with acute Crohn's disease as it has been shown to be effective in induction of remission, it improves nutritional status, and it reduces the deleterious effects of corticosteroids on growth [108].

Oral nutritional supplements or enteral nutrition can be recommended in patients with Crohn's disease in remission, if undernutrition cannot be treated sufficiently by dietary counselling [101]. In severely malnourished children and adolescents with IBD, refeeding syndrome can occur, and electrolyte imbalances should be monitored and treated [109]. All IBD patients in remission should undergo counselling by a dietitian as part of the multidisciplinary team approach to improve nutritional therapy and to avoid malnutrition and nutrition‐related disorders.

#### 2.2.6 Statement

Malnutrition is a common complication of cholestasis and cirrhosis in childhood and adolescence. We recommend avoiding prolonged periods of fasting and to use dietary supplements such as medium chain triglycerides oil or more intensive nutritional support with enteral or parenteral nutrition and supplementation of micronutrient deficiencies, especially fat‐soluble vitamins.

Children and adolescents with chronic liver disease are at increased risk of malnutrition. Malnutrition is a common complication of cholestatic and end‐stage liver diseases [64]. Initially, malnutrition in children and adolescents with cholestasis may be due to maldigestion and malabsorption of nutrients, along with an increased metabolic demand. In children and adolescents with end‐stage liver disease requiring transplantation, optimized pre‐transplant nutrition may hasten post‐transplant recovery while simultaneously decreasing complications.

Patients with chronic liver diseases, especially cholestasis, often have deficiencies of protein, essential fatty acids and fat‐soluble vitamins [110]. Protein requirements are typically increased due to protein loss (via stool, urine or into the interstitial space), increased amino acid oxidation, and poor nutritional status [111]. Essential fatty acid deficiencies can be secondary to fat malabsorption, inefficient elongation of essential fatty acid precursors by dysfunctional hepatocytes, and enhanced peroxidation of lipids, but can also be iatrogenic, particularly when diets high in medium chain tryglicerides (MCT) and low in long‐chain triglycerides are used [112]. Unabsorbed free fatty acids bind to dietary calcium leading to gastrointestinal calcium losses, contributing to metabolic bone disease and oxalate nephrolithiasis [64]. Nutrition support can be optimized using MCT oil, nasogastric feeds or total PN.

#### 2.2.7 Statement

Elderly individuals are more likely to develop certain diseases, or to use certain medications, causing malabsorption. These diseases include, among others, exocrine pancreatic insufficiency, vascular disease, and diabetes mellitus.

Overt malabsorption should not be considered per se as a part of the physiological deterioration of gastrointestinal function related to ageing [113, 114, 115]. However, elderly people frequently show increased intestinal permeability even in the absence of gastrointestinal symptoms and this may constitute a risk factor for malabsorption and malnutrition [115, 116]. Of note, the risk of malabsorption is increased in the elderly, as a result of diseases that have an increased prevalence in this age group, such as EPI, enteropathies (especially drug‐induced), small bowel ischaemia, diabetes, lactose malabsorption, and certain infections, such as Whipple's disease. Furthermore, in the elderly, the use of medications that may cause malabsorption and the exposure to surgery that can impact on the absorptive process are more common [117, 118, 119].

Among the most common causes of malabsorption in the elderly, lactose malabsorption, EPI and CD are worth mentioning, these two latter contributing together to up to 60% of cases (up to 30% of cases for each one, respectively) [120, 121]. The diagnosis of malabsorption in the elderly can be challenging because of the non‐specific and subtle symptoms, with a consequent diagnostic delay. Sometimes, anorexia, nausea, diarrhoea, abdominal pain, bloating, and excessive flatus can be the only manifestations of malabsorption [121, 122].

#### 2.2.8 Statement

Vitamin B12 deficiency is common among the elderly, mainly due to the high prevalence of autoimmune atrophic gastritis and food‐cobalamin malabsorption syndrome in this age group. Similarly, folate deficiency is common, due to malabsorption combined with low folate intake. Folate and vitamin B12 deficiency are a reversible cause of neuropsychiatric symptoms and haematological alterations in the elderly.

Vitamin B12 deficiency is common in the elderly, with a prevalence ranging from 5% to 20%, depending on the used cut‐off, with an increasing prevalence with age [123, 124, 125]. The most common causes of vitamin B12 deficiency in the elderly are food‐bound cobalamin malabsorption (> 60% of cases), with a prevalence of 10%–30% in people over 50‐year‐old, characterized by an impaired ability to separate cobalamin from food or from intestinal transport proteins [126], and autoimmune atrophic gastritis, leading to intrinsic factor deficiency with consequent vitamin B12 malabsorption [38]. Parenteral supplementation of the vitamin is necessary only in case of severe anaemia or neurological alterations [38]; oral crystalline vitamin B12 may be sufficient in less severe cases [127, 128]. Other causes, often concomitant, may contribute to vitamin B12 deficiency in the elderly. The high prevalence of autoimmune atrophic gastritis with the loss of the parietal cells, SIBO and iatrogenic hypochlorhydria due to chronic PPI use may also aggravate the deficit [129, 130, 131, 132, 133, 134]. Screening for vitamin B12 is important in the elderly, as some related neurological abnormalities could be reversed after supplementation, even if it does not impact on the progression of dementia [135, 136, 137].

Folate malabsorption is common in the elderly and may be related to hypochlorydria [137]. Low folate absorption, associated to low dietary intake, is responsible for low folate levels in the elderly, and associated with neurologic symptoms such as mild cognitive impairment, dementia (particularly Alzheimer's disease), depression, and macrocytic anaemia. However, the ability of folate supplementation to reverse or slow cognitive decline is still topic of debate [138].

#### 2.2.9 Statement

Women with a history of bariatric surgery should be screened for nutritional deficiencies before, during, and after pregnancy and they should plan a pregnancy at least 1 year after bariatric surgery to reduce the risk of adverse pregnancy outcomes. Women with infertility and obstetric complications of apparently unknown cause should be screened for coeliac disease, vitamin B12 deficiency, and autoimmune atrophic gastritis.

Low levels of micronutrients are common in pregnant women after bariatric surgery.

On this base, women should be screened before conception or at the first antenatal visit, for blood cell count, ferritin, iron, vitamin B12, folate, thiamine, vitamin A, calcium, and vitamin D. Testing should be repeated at least once per trimester and during the post‐partum period if breastfeeding, as micronutrients could be depleted during pregnancy, thus supplementation could be necessary [139, 140, 141, 142].

In contrast, patients undergoing merely restrictive surgery (i.e., gastric banding) showed no increase in the rate of small for gestational age infants [141, 142, 143].

Women with early pregnancy loss should be screened for vitamin B12 deficiency as low levels of low vitamin B12 and hyperhomocysteinemia are individual risk factors for early pregnancy loss in a case‐control study and a meta‐analysis of observational studies, with an increased risk when both the alterations are present in the same subject (OR = 4.98, *P* = 0.002) [144]. Autoimmune atrophic gastritis, in small reports, was a cause of vitamin B12 deficiency in pregnant women having adverse pregnancy outcomes [43].

Infertility and obstetric complications are known clinical manifestations of CD, due to malabsorption of folic acid, vitamin B12, fat‐soluble vitamins, iron, and zinc [145], but also due to a direct effect of anti‐transglutaminase antibodies [146]. The role of CD in unexplained infertility has been largely evaluated in the literature, but the evidence supporting a higher prevalence of CD in women suffering from infertility is not conclusive and there is high variability among studies.

CD may not only be related to infertility, but also to adverse pregnancy outcomes ‐including miscarriage, intrauterine growth retardation, low birth weight, small for gestational age, stillbirth, preterm birth‐that could be up to 4 times more frequent in women with CD compared to healthy controls [147, 148, 149, 150]. Conversely, patients with history of recurrent miscarriage or intrauterine growth retardation had a higher risk of CD compared to the general population [151]. The early diagnosis of CD and the tempestive introduction of gluten free diet reverse the negative effects associated to untreated CD, including recurrent pregnancy loss [152, 153, 154, 155]. However, more studies are needed in this field before specific recommendations could be drawn.

### 2.3 What Is a Reasonable Algorithm Which Combines Screening Tests, Laboratory and Endoscopic Evaluations and Radiological Tests?

#### 2.3.1 Statement

**FIGURE 1:**
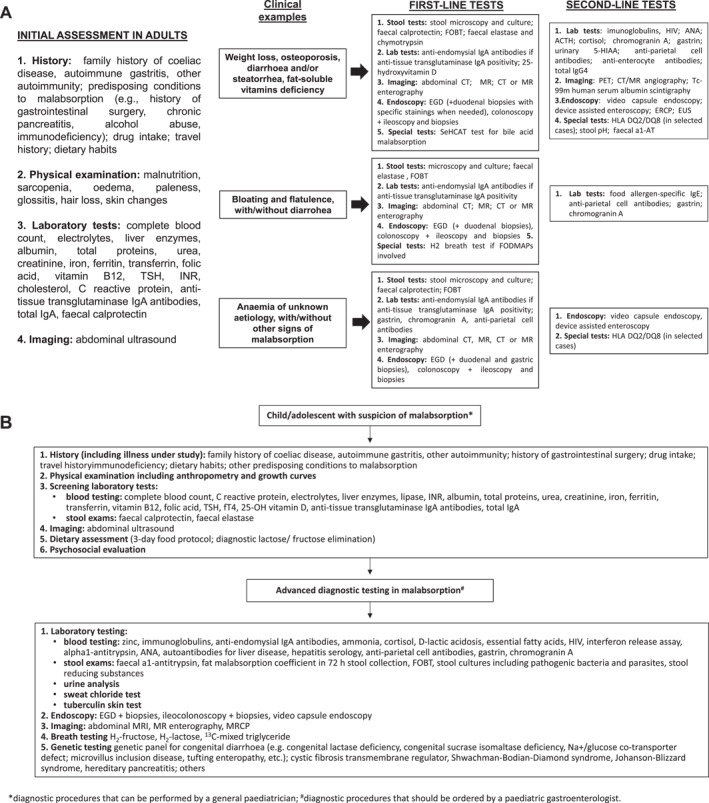
(A) Schematic algorithm for the diagnosis of malabsorption in adults. (B) Schematic algorithm for the diagnosis of malabsorption in children and adolescents. The algorithm should be considered as expert‐based, and the order of the examinations may vary depending on specific clinical situations and local test availability. Indeed, if a specific cause of malabsorption is highly likely depending on patient's history, the order of testing will depend on this. For example, overt malabsorption in a patient with a first‐degree family history of coeliac disease (CD) should prompt further testing for CD, while leaving other tests as a second line in case CD is not confirmed. Similarly, in a patient with a single nutrient deficiency, such as vitamin B12 with pernicious anaemia, autoimmune gastritis would be the most likely diagnosis. The initial assessment, both in adults and children, should be made by any physician, while second‐level testing should be recommended by specialist physicians. For adults, three different illustrative clinical examples are provided based on the presenting features. Indeed, not all examinations are necessary in every case, and the order may vary, as previously mentioned. A universal algorithm that fits every clinical picture dominated by malabsorption does not exist; specific guidelines should be followed for individual diseases. The same principle is applied to the paeditric setting. a1‐AT, alpha 1‐anti‐trypsin; CT, computer tomography; EGD, esophagogastroduodenoscopy; ERCP, endoscopic retrograde cholangiopancreatography; EUS, endoscopic ultrasound; FOBT, faecal occult blood test; INR, international normalized ratio; MR, magnetic resonance; PET, positron emission tomography; US, ultrasound.

Initial assessment of each patient with suspected malabsorption syndrome should include a detailed history, physical examination, screening laboratory tests, and abdominal ultrasound in selected patients. In patients with clinically‐ and laboratory‐proven malabsorption syndrome, a stepwise approach is recommended to determine its cause, with diagnostic tests grouped according to availability, frequency of disease in the population, invasiveness, and cost (Figure 1A for adults and 1B for children).

Establishing the correct diagnosis in patients presenting with malabsorption syndrome can be a challenging task even for experienced clinicians. One of the reasons is that several pathophysiological mechanisms may exist concurrently, and malabsorption syndromes can be caused by a large number of diseases with very variable clinical manifestations. This is also a reason why it is difficult to postulate one universal, useful, and well‐arranged algorithm on how to proceed in such patients. Published literature and research focussing on the most appropriate approach in patients with malabsorption are scarce. In the reviewed literature only 3 diagnostic algorithms of malabsorption syndromes were found, all of them as part of systematic reviews (including 1 algorithm for chronic diarrhoea only) [156, 157, 158].

The goal of diagnosing malabsorption is to establish a proper diagnosis quickly, with a minimum of invasive diagnostic procedures and costs [159]. The most important step is to discuss the patient's history, signs and symptoms in detail, to perform a physical examination, make routine screening laboratory tests and an abdominal ultrasound [159]. The outcome of these tests is helpflul in raising the suspicion of malabsorption and its aetiology, and in choosing proper laboratory, functional, endoscopic and imaging tests as next evaluations. It has been suggested that with these first step an expert physician can correctly diagnose 70%–80% of malabsorption syndromes [159]. The sequence of additional tests should be followed according to results of initial assessments and availability of diagnostic methods. Figure 1A suggests an algorithm according to different clinical presentations in adults, while Figure 1B focuses on the paediatric setting. In Figure 1A, we provide some illustrative examples of potential first‐ and second line tests, but the overall diagnostic process may differ depending on the specific case.

## 3 Nutritional Supplementation, Treatment Goals, Supportive Care

### 3.1 When Is Nutritional Supplementation Needed and Which Kind?

#### 3.1.1 Statement

Many patients with malabsorption have enough remaining absorptive function to allow management of nutritional support by the oral or enteral route. However, some patients may develop intestinal failure, defined as a reduction of intestinal function below the minimum requirement for maintenance of body function, composition, and homoeostasis, thus requiring intravenous supplementation to maintain health and/or growth.

The severity of malabsorption syndrome can vary, and many patients have sufficient absorptive capacity to maintain health, including growth in children. Patients with persistently reduced intestinal function that can still be managed with dietary modification, oral or enteral supplementation are considered to have intestinal insufficiency [160, 161].

If reduced absorptive capacity compromises maintenance of health and/or growth and oral or enteral diet is not sufficient, the patient is considered as having intestinal failure and will require intravenous supplementation (macronutrients, micronutrients, water, electrolytes) [162]. Both criteria must be simultaneously present to diagnose IF, that is, decreased absorption of macronutrients and/or water and electrolytes due to a loss of gut function, and the need for intravenous supplementation.

Congenital or acquired malabsorptive syndromes due to extensive small bowel mucosal disease are included as one of the five pathophysiological conditions that may result in IF [163].

The severity of IF due to malabsorptive syndromes is based on the content and the volume of intravenous requirements. In patients requiring only fluid and electrolyte replacement IF is less severe than in patients requiring PN containing macronutrients. As the volume of the PN, calculated on a weekly basis, increases, the severity of intestinal failure also increases [162].

#### 3.1.2 Statement

Patients with malabsorption and malnutrition, or at risk of developing malnutrition, require dietetic advice. Patients who cannot meet their nutritional requirements with conventional foods will require medical nutrition therapy. Oral nutritional supplements are the first step when medical nutrition is indicated in malabsorption syndromes. Enteral nutrition should be considered for patients in whom oral feeding is insufficient. Parenteral nutrition should be reserved for patients with intestinal failure.

Nutritional support should be considered in patients who are malnourished or are at risk of developing malnutrition. Identifying the risk of malnutrition can be done using any validated screening tool [164]. Most malnutrition screening tools assess, among others, involuntary weight loss, low body mass index, reduced food intake or disease burden. Once nutritional risk has been identified, diagnostic criteria for malnutrition include at least one of the phenotypic criteria, that is, weight loss, low BMI or reduced muscle mass, and at least one of the aetiological criteria, that is, reduced food intake/assimilation, or inflammation (both acute and chronic). Therefore, patients with malabsorptive syndromes who are at risk of malnutrition require monitoring and identification of weight loss, low BMI or loss of muscle mass.

Dietary counselling is the first step to help patients to meet their nutritional requirements, with the aim of improving nutrient intake from normal foods. Dietary strategies may include food modification (in terms of macronutrient content, meal distribution, or texture change) and food fortification. Patients with malabsorption often require a high‐protein, high‐calorie and low‐to‐modified fat diet to minimize steatorrhoea.

Patients who are unable to meet their nutritional requirements with dietary advice will require medical nutrition therapy. Oral nutritional supplements (ONS) are used when the patient can swallow safely and the gastrointestinal tract is functional, and they can facilitate the treatment of nutritional deficiencies and the repletion of body tissues [63, 162]. Severe anorexia, swallowing problems or obstructive and motility problems of the gastrointestinal tract may limit the use of ONS. For people who have failed, or are unlikely to respond to oral nutrition therapy, enteral tube feeding should be considered. It is essential to have an accessible gastrointestinal tract, adequate gastrointestinal motility and sufficient absorptive capacity.

Finally, PN should be given to patients with IF that cannot be managed by the oral route alone. PN may have to cover nutritional needs partly or completely. The main reason for the use of NP in patients with malabsorption is severe impairment of the absorptive capacity [162, 165].

#### 3.1.3 Statement

Patients with malabsorption may develop micronutrient deficiencies. The micronutrient(s) affected will depend on the pathophysiological mechanism of the malabsorptive syndrome, and the segment(s) of the gastrointestinal tract affected. In malabsorptive syndromes, micronutrient deficiencies should be screened, and any deficiency should be properly corrected. Micronutrient supplementation shall be provided by the oral or enteral route, if this can be done safely and effectively, depending on the pathophysiological mechanisms underlying malabsorption. If the oral or enteral route is not safe or effective, then other routes may be considered (e.g., intramuscular, sublingual, parenteral).

#### 3.1.4 Statement

Monitoring of fat‐soluble vitamins (A, D, E, K), and supplementation in case of deficiency, should be performed regularly in disorders associated with fat malabsorption, such as cystic fibrosis and other causes of exocrine pancreatic insufficiency, cholestatic liver disease, short bowel syndrome, congenital intestinal lymphangiectasia, abetalipoproteinemia, and in patients with malabsorption due to bariatric surgery.

#### 3.1.5 Statement

Monitoring of some vitamins of group B (e.g., B1, B6, folate), and supplementation in case of deficiency should be performed regularly in conditions associated with malabsorption. Vitamin B12 should be monitored in all patients with compromised absorption due to intrinsic factor deficiency (e.g., total gastrectomy, autoimmune atrophic gastritis) or distal ileal disease (e.g., Crohn's disease or previous ileal resection). Monitoring of some minerals (e.g., Fe, Mg, Se, Zn, Cu), and supplementation in case of deficiency, should be carried out regularly in chronic malabsorption.

A number of micronutrient deficiencies have been identified in patients with malabsorptive syndromes, including both essential trace elements and vitamins [166].

Dietary iron absorption occurs mainly through enterocyte cells on the duodenum and upper jejunum of the small intestine. Causes of iron malabsorption include premucosal conditions that result in maldigestion of iron due to improper mixing of gastrointestinal enzymes and bile with digestive secretions in post‐gastrectomy, chronic pancreatitis, cystic fibrosis, pancreatic resection, Zollinger‐Ellison syndrome, or terminal ileal resection. Mucosal malabsorption of iron occurs in CD, tropical sprue, Crohn's disease, autoimmune gastritis, and other conditions. Postmucosal malabsorption of iron arises due to impaired nutrients transport for example, intestinal lymphangiectasia and macroglobulinemia [167].

Copper absorption occurs in the stomach and small intestine, primarily in the duodenum. Copper deficiency may be seen in bariatric surgery and other abdominal surgery that excludes the duodenum, and in patients receiving long‐term enteral nutrition via a jejunostomy tube [168]. Zinc malabsorption may occur in patients with primary acrodermatitis enteropathica, short bowel syndrome (SBS), bariatric surgery, cystic fibrosis, chronic pancreatitis, IBD or a diet rich in phytate [169].

Thiamine (vitamin B1) is rapidly absorbed in the jejunum and ileum. Reduced gastrointestinal absorption may occur in SBS or after bariatric surgery. Riboflavin (vitamin B2) is absorbed mainly in the proximal small intestine. Riboflavin is also produced by the microflora of the colon. Patients with SBS or CD are at risk of malabsorption [170]. Malabsorption of niacin may occur in malabsorptive syndromes with prolonged diarrhoea. Regarding biotin (vitamin B7), conditions at risk of developing deficiency include malabsorption associated with malabsorptive syndromes such as Crohn's disease and colitis, SBS and CD [171].

Folates (vitamin B9) are absorbed in the duodenum and jejunum. Causes of folate deficiency include intestinal malabsorption syndromes such as CD, IBD, post‐bariatric surgery, post‐gastrectomy, chronic IF, and autoimmune atrophic gastritis [170, 171, 172].

Absorption of cobalamin from food requires normal function of the stomach, pancreas, and small intestine; patients with intestinal resection or surgical reconstructions are therefore at a high risk of B12 deficiency [173]. The most common causes of B12 deficiency are autoimmune atrophic gastritis causing pernicious anaemia [38] and food‐bound cobalamin malabsorption. Cobalamin deficiency has become a common problem in bariatric surgery patients [172], and also in gastrointestinal surgery such as gastrectomy or pancreatoduodenectomy [174]. The intramuscular route of administration of B12 should be used in patients with a history of total gastrectomy, extensive ileal resection or persistent malabsorptive disease. Intramuscolar injections of 1000–2000 mcg of cobalamin every 1–3 months are suggested [175]. Patients with food‐bound cobalamin malabsorption should receive lifelong supplementation either as a daily dose of 350 μg of cobalamin orally or as intramuscolar injections of 1000–2000 mcg of cobalamin every 1–3 months. An oral, sublingual formulation of vitamin B12 is now also available.

Fat‐soluble vitamins including vitamin A and carotenoids follow the absorption of lipids in the gastrointestinal tract, and their absorption presumably occurs in the upper half of the small intestine [176]. Measurement of serum retinol and retinyl esters (if available) should be considered in patients being investigated for malabsorption. In conditions causing fat malabsorption, prevention of deficiency with oral supplements may be considered.

Patients with fat malabsorption due to either inflammatory diseases, SBS or cystic fibrosis are at risk for inadequate intake of fat‐soluble micronutrients. In fat malabsorption, low plasma lipid levels are associated with low plasma vitamin E levels, so a low ratio of plasma α‐tocopherol to plasma lipids (< 0.8 mg/g total lipid) is the most accurate indicator of vitamin E deficiency in adults [177].

The most common causes of vitamin K deficiency are conditions involving fat malabsorption (such as CD, cystic fibrosis, SBS, and chronic pancreatitis) [178], malnutrition, antibiotic treatment and anticoagulant treatments (warfarin). Vitamin K status may be measured in patients at risk, including those with symptoms due to steatorrhoea.

#### 3.1.6 Statement

The type of oral nutritional supplement depends on the aetiology and severity of the malabsorption syndrome. In general, polymeric formulae are preferred because of their lower osmolarity and better palatability than oligomeric formulae. However, some patients with malabsorptive syndromes require some degree of hydrolysis of macronutrients to allow the absorption of the oral nutritional supplement, therefore, oligomeric formulae may be a better choice. In patients with severe malabsorption who cannot be managed with oligomeric formulae, elemental formulae are recommended.

In patients with IBD who require supportive medical nutrition therapy, ONS are indicated as a first step supportive therapy in addition to a normal diet. ONS or EN are recommended in patients with Crohn's disease in remission if malnutrition cannot be adequately addressed by dietary counselling [63]. Primary nutritional therapy in the form of exclusive enteral nutrition, usually by the oral route, is recommended as the first‐line therapy to induce remission in children and adolescents with mild active Crohn's disease, and as adjunctive therapy in adults [63].

Several studies have evaluated the efficacy and tolerability of different types of formula in mild active IBD, both in the form of ONS and enteral nutrition [179]. As one of the main reasons for stopping enteral nutrition was unpalatability of the diet, polymeric formulae with moderate fat content are preferred as a first supportive therapy. There is no evidence that an oligomeric diet is superior to a standard formula.

In patients with malabsorption due to chronic pancreatitis or EPI, semi‐elemental formulae containing MCT may be required in addition to enzyme replacement therapy [180].

#### 3.1.7 Statement

When oral nutrition is not sufficient, enteral nutrition should be advised. The type of tube (nasoenteral or feeding ostomy) and the route (gastric or jejunal) to deliver enteral nutrition will depend on the specific clinical situation. For short‐time enteral support, a nasogastric or nasojejunal tube may be preferred, whereas a feeding ostomy is recommended for longer periods. The gastric route is preferred because it is more physiological and provides a better tolerance to volume. The jejunal route is indicated in patients with gastroparesis or associated gastroesophageal reflux.

In patients with malabsorptive syndromes requiring medical nutrition therapy, enteral nutrition should be considered if oral feeding is not sufficient. The most appropriate route for outpatient nutritional support depends on the functioning, accessibility and digestive and/or absorptive capacity of the gastrointestinal tract [181]. If enteral nutrition is required for a short period of time (4–6 weeks), nasogastric tube feeding is the preferred route. If long‐term enteral nutrition is required, a feeding ostomy (endoscopic or radiologically inserted gastrostomy) should be used. Gastric placement of the tube allows the bolus infusion of enteral nutrition, which is considered more physiological. In some cases, continuous infusion through a pump may be required, depending on clinical need, safety and the level of precision required.

The jejunal route is a suitable approach in cases of gastroduodenal motility disorders (mainly gastroparesis) or high risk of aspiration, as in gastroesophageal reflux. In these cases, a post‐pyloric feeding may help to prevent aspiration. Patients with chronic pancreatitis who require enteral nutrition and who have pain, delayed gastric emptying, persistent nausea and vomiting and gastric outlet obstruction should be fed via the jejunal route [182]. In patients with gastrectomy, the jejunal route is the only possible choice for enteral nutrition.

#### 3.1.8 Statement

Parenteral nutrition should be reserved for patients with intestinal failure, who cannot maintain the nutritional status or growth via the oral/enteral route. The type of parenteral support should be tailored, and may include intravenous fluids and/or electrolytes, complementary parenteral nutrition or total parenteral nutrition, depending on the degree of malabsorption and malnutrition.

Intravenous nutritional supplementation is an essential therapy required to maintain health and/or growth in patients with intestinal failure [162]. Malabsorptive syndromes are a common cause of intestinal failure, usually due to extensive mucosal disease or resection. The severity of intestinal failure shall be categorized according to the type and volume of intravenous supplementation, which may consist of intravenous fluids alone or ofparenteral nutrition formula [183]. The composition of the parenteral formula should be tailored in order to avoid metabolic complications associated with parenteral nutrition.

### 3.2 What Are the Treatment Goals and How Shall Treatment be Monitored?

#### 3.2.1 Statement

Nutritional status should be monitored regularly in patients with malabsorptive diseases, as one of the goals of treatment is to maintain or improve nutritional status. In children with malabsorptive diseases, normal growth and development should be included as a treatment goal. Monitoring of nutritional status should include weight, height, BMI, muscle mass and muscle function, and laboratory tests when applicable.

Patients with malabsorptive syndromes are at high risk of malnutrition, as reduced food assimilation is one of the main criteria for the diagnosis of malnutrition [184]. Therefore, the goals of therapy in adults will be to prevent weight loss, improve BMI and/or increase muscle mass. In children, the main goal is normal growth and development.

Sarcopenia, defined as loss of muscle mass accompanied by loss of muscle strength, is common in patients with IBD. A systematic review of 19 body composition studies involving 926 IBD patients revealed a low fat‐free mass in 28% of Crohn's disease patients [185]. Identifying and treating sarcopenia is an important goal in the management of IBD, due to the impact on quality of life, prognosis and outcome after surgical, biological or immunomodulatory treatment [186].

Muscle mass should be assessed by a validated technique such as bioelectrical impedance, DEXA, CT scan or muscle ultrasound, or by anthropometric measures such as calf or mid‐upper arm circumference, using validated ethnic and gender‐specific cut‐off values where available [187].

### 3.3 Which Supportive Medical or Surgical Treatments May be Appropriate?

#### 3.3.1 Statement

Antimotility drugs (e.g., loperamide, diphenoxylate, codeine, opium tincture, octreotide) may be useful in patients with severe malabsorption such as intestinal insufficiency, to reduce gastrointestinal fluid losses. Loperamide is preferable to diphenoxylate because of the lack of central nervous system effects. In severe cases of diarrhoea, a combination of loperamide and codeine may be useful. The dose of the anti‐motility agents should be adjusted and escalated in a stepwise manner interval until maximum benefit is observed, adverse events occur, or the recommended maximum dose is reached.

The use of antimotility drugs, mainly loperamide and codeine, is widespread in patients with IF or intestinal insufficiency and aims at reducing water and electrolyte losses in the stool. The use of loperamide should be preferred to opiate drugs (codeine, opium, diphenoxylate), because of the lack of central nervous system side effects (mainly sedative effects). Loperamide inhibits the peristaltic activity of the small intestine and prolongs intestinal transit time, thus increasing the time for water and sodium absorption. The optimal dose and tolerability of the antimotility drugs may be tailored and adjusted until maximum benefit is observed, adverse events occur, or the recommended maximum dose is reached. In general, loperamide, 4 mg given three to four times a day is recommended [165]. Small, randomized placebo‐controlled trials of loperamide have been conducted, but mainly in patients with an ileostomy or ileocaecal resection and diarrhoea. In general, treatment reduced faecal wet weight output by 15%–30%.

The efficacy of loperamide and codeine has been studied in small trials as an adjunct to bile acid sequestrant therapy in patients with diarrhoea due to bile acid malabsorption [188].

Finally, in case of severe, refractory diarrhoea, octreotide has also been proposed, due to its anti‐motility and anti‐secretory effect [189].

#### 3.3.2 Statement

There is currently no evidence to suggest that probiotics have a role in inducing or maintaining remission in children or adults with Crohn's disease, or in treating other causes of malabsorption.

There is insufficient data to recommend the use of probiotics for the treatment of active Crohn's disease. Two small studies have evaluated the efficacy of probiotic treatment (*Lactobacillus rhamnosus strain GG* [190] and *Biffidobacteroum longum* [191]) as adjunctive therapy for mild‐to moderate Crohn's disease in adult patients. There was no evidence of a difference between the use of probiotics and placebo in inducing remission in at 6 months [192]. Some studies have focussed on the role of probiotics in maintaining Crohn's disease remission, and two meta‐analyses suggest that probiotics are not a therapeutic option for maintaining remission [193, 194].

#### 3.3.3 Statement

There is insufficient data to recommend the use of prebiotics, such as fructans (e.g., inulin), non‐digestible polysaccharides, galacto‐oligosaccharides (GOS), oligosaccharides, or fructo‐oligosaccharides (FOS) as a treatment for patients with malabsorption syndrome.

Prebiotics are non‐digestible, fermentable food ingredients that alter the composition and/or activity of gastrointestinal bacteria and confer benefits to the host. The main groups of prebiotics are fructans, which include inulin and fructo‐oligosaccharides (FOS), galacto‐oligosaccharides (GOS), lactulose, resistant starch, glucose‐derived oligosaccharides such as polydextrose, and pectin oligosaccharides (POS) [195].

Two randomized clinical trials evaluated oligofructose‐enriched inulin at 15–20 g/day and found no benefit in inducing clinical remission in adults with Crohn's disease [196, 197].

One study evaluated the effect of 10 g of oligofructose‐enriched inulin versus placebo on intestinal permeability in a group of children with CD on gluten‐free diet. After 12 weeks, prebiotic supplementation had no significant effect on barrier integrity in this group of patients [198].

Available data suggest the possible beneficial effect of butyrate supplementation in patients with IBD. Butyrate is one of the major short‐chain fatty acids that are the final product of saccharolytic fermentation of complex and non‐digestive polysacchaides by anaerobic bacteria. Previous small studies demonstrated that enteric‐coated butyrate effectively reduced ileocaecal inflammation and maintained clinical remission in Crohn's disease patients [199]. A new oral formulation of microencapsulated sodium‐butyrate that can reach the colon is available in some European countries for the oral administration. In a pilot study, lipophilic microencapsulated sodium butyrate treatment showed enrichment of butyrogenic colonic bacteria in IBD patients [200]. Further studies are needed to assess the clinical impact of oral administration of exogenous butyrate effect on clinical activity and mucosal healing in IBD patients.

#### 3.3.4 Statement

There are insufficient data to recommend routine oral rehydration therapy in patients with malabsorption. Patients with acute intestinal failure due to short bowel syndrome may benefit from sodium‐rich fluids to improve net intestinal absorption.

Oral rehydration therapy, originally developed to treat severe dehydration from diarrhoea caused by cholera, has been reconsidered and used in patients with SBS to optimize the water absorption and reduce the intestinal losses. Even with reduced surface area, the sodium‐glucose cotransport system is unaffected and is able to efficiently absorb fluid in the presence of sodium and glucose in the bowel. The presence of glucose in the lumen stimulates the active transport of both glucose and sodium across cell membranes via the sodium‐glucose cotransporter. This creates an osmotic gradient, leading to water absorption and a reduction in stool output [201]. Patients with acute intestinal failure due to SBS, especially those without colon, should use oral rehydration fluid as their main source of oral hydration [202]. It is also recommended that patients sip the fluids, and separate intake of liquids from intake of solids to prevent the rapid transit of solids and to improve the absorption [203, 204]. In IBD patients with high output jejunostomy, fluid intake should be adjusted accordingly (decrease hypotonic fluid and increased saline solution) [63]. There are insufficient data to recommend the use of oral rehydration solutions in other malabsorptive syndromes to attempt to reduce intestinal losses.

#### 3.3.5 Statement

In patients with short bowel syndrome and high faecal output, the use of H_2_‐receptor antagonists or proton pump inhibitors may be effective in reducing faecal wet weight and sodium excretion.

After bowel resection, gastric hypergastrinemia and hypersecretion occur, contributing to total faecal water and electrolyte losses. In addition, the associated hyperacidity may denature pancreatic enzymes and impair bile salt function, which may further aggravate malabsorption. Treatment of hypersecretion in patients with SBS with H2‐receptor antagonists or proton pump inhibitors can be used to reduce faecal wet weight and sodium excretion, especially during the first 6 months after surgery and especially in those SBS patients with a faecal output greater than 2 L/day [162]. Two studies demonstrated their efficacy in reducing ostomy output in SBS patients, with reductions in faecal wet weight and sodium excretion in the range of 20%–25% [205, 206]. Long term treatment with an H_2_ receptor antagonist or a proton pump inhibitor should be decided on a case by case basis, taking into account safety issues related to vitamin B12 deficiency, hypomagnesaemia, hypokalemia and impairment in calcium absorption [207].

#### 3.3.6 Statement

The use of glucagon‐like peptide 2 (GLP‐2) analogues (e.g., teduglutide) is recommended for patients with intestinal failure due to short bowel syndrome who require parenteral fluids or nutrients after the period of intestinal adaptation, in order to reduce or withdraw parenteral support and improve quality of life. Patients should be carefully informed of the potential benefits and risks associated with this treatment. The efficacy of treatment with a GLP‐2 analogue should be assessed according to standardized protocols which include measurement of fluids, electrolytes, and nutritional status.

In patients with IF due to SBS, intestinal growth factors should be considered for patients requiring parenteral nutrition/parenteral fluids who are stable after postoperative intestinal adaptation of usually 12–24 months after the last intestinal resection [162]. GLP‐2 is a trophic hormone secreted by intestinal L‐cells of the lower small and large intestinal mucosa in response to the presence of nutrients in the gut lumen. GLP‐2 increases the intestinal capacity to absorb nutrients by promoting intestinal crypt cell proliferation, inhibiting enterocyte apoptosis and gastric acid secretion, decreasing small intestinal motility, and increasing mesenteric blood flow [208]. Teduglutide is a recombinant GLP‐2 receptor agonist, resistant to degradation by dipeptidyl‐peptidase IV and thus with an extended half‐life compared to native GLP‐2 and is the only recombinant analogue of GLP‐2 approved in the United States and Europe for the treatment of patients with SBS‐chronic IF. Other GLP‐2 receptor agonists with longer half‐life are under clinical development.

Three randomized controlled trials [209, 210, 211], one controlled trial [212] and other cohort studies [213, 214, 215, 216] demonstrated the safety, efficacy and tolerability of teduglutide treatment. Teduglutide treatment reduces PN dependence in adult patients with SBS, after a period of intestinal adaptation following surgery. Efficacy of the intestinal growth factor treatment is defined as a 20% stable reduction in intravenous volume from baseline. Prior to start the treatment, all patients have to undergo colonoscopy (if remnant colon and/or rectum), abdominal ultrasound, and gastroscopy to assess for the presence of polyps and to rule out neoplastic disease. As GLP‐2 stimulates crypt cell proliferation and exerts anti‐apoptotic effect, survey for the risk of intestinal neoplasia is mandatory for patients receiving treatment with GLP‐2 analogues. In children, a systematic review of 14 studies concludes that teduglutide appears to be safe and effective in reducing PN requirements and improving enteral autonomy in the paediatric population [217]. However, further studies are needed to understand long‐term efficacy and potential complications. Patients with chronic IF due to SBS should be carefully informed of the potential benefits and risks associated with intestinal growth factor treatments; the information should address the likelihood of reducing the need for or the weaning from home PN, the likelihood of improving quality of life, the expected duration of treatment, the expected effects after discontinuation of the treatment, the potential adverse effects and risks of the treatment, the cost‐effectiveness of the treatment, and the need for careful and regular monitoring. Treatment‐emergent adverse events are very common and mostly mild or moderate in severity. The most common are abdominal pain, nausea and abdominal distension, which are more common early in the course of treatment and decrease over time. The risk of new gastrointestinal, hepatobiliary and pancreatic benign or malignant neoplasms and the consequent need for appropriate follow up screening should be clearly highlighted [218].

Body weight and composition, electrolyte balance and renal function should remain stable despite the reduction in IV supplementation. Careful monitoring of the treatment by an expert multidisciplinary team is mandatory, performed by and according to standardized protocols. Patients should be aware of the high likelihood of a lifelong treatment duration and a return to baseline IVS requirements if treatment is discontinued.

#### 3.3.7 Statement

Restoration of intestinal continuity in patients with a stoma, whenever possible, and non‐transplant surgery focussing on bowel lengthening procedures may be recommended in selected patients with short bowel syndrome to increase mucosal surface area.

In patients with SBS who have an ostomy, bowel continuity should be restored whenever possible, to reduce home PN dependency. Once the patient is stabilized, reconstruction of gastrointestinal continuity should be prioritized whenever feasible by ostomy reversal and recruitment of distal bowel [162]. This also includeds patients who develop diversion colitis. Restoration of bowel continuity may improve PN‐induced chronic cholestasis by reducing the need for PN [219]. Surgical intervention to restore continuity may be individualized and only performed by experienced surgeons in specialized interdisciplinary units.

Bowel lengthening procedures may be considered in selected patients [220]. Segmental bowel dilatation with poor peristalsis is a common finding in paediatric patients with SBS. Tapering techniques without loss of surface area are achieved by both with the longitudinal intestinal lengthening and tailoring (LILT), and the serial transverse enteroplasty (STEP) procedure. The choice of lenghtening procedure seems to be related to the experience of the surgical team, with little difference between the two techniques regarding the enteral autonomy achieved, the improvement of liver cholestasis and the complications rate [221, 222]. Patients with SBS should be managed with a multidisciplinary approach to optimize intestinal rehabilitation and overall patient outcome.

In patients with Crohn's disease who develop strictures, rather than performing small bowel resection, strictureplasty should be preferred when feasible, in order to avoid short bowel [223].

Finally, small bowel or multi‐visceral transplantation have also been performed in few, very severe and refractory cases of SBS and IF. However, the need for longterm immunosuppression, the technical difficulties in performing this surgery, and potential complications are major limitations [224].

## 4 Primary Care Perspective

### 4.1 Statement

Primary care physicians can suspect malabsorption syndromes by recognizing a variety of symptoms and signs including, among others, chronic diarrhoea, significant weight loss, anaemia, short stature, steatorrhoea and discoloured stool, abdominal pain, nausea, bloating, flatulence, fatigue.

It is crucial to highlight the importance of considering the possibility of malabsorption syndrome early in the consultation process of patients presenting with gastrointestinal symptoms and/or predisposing conditions, and to refer the patient for further evaluation, if necessary. Despite the probably high prevalence of malabsorption syndromes in family medicine, they can be easily missed due to their broad spectrum of manifestations, as in the case of CD [225, 226, 227], which is also one of the most common causes of generalized malabsorption syndrome or of isolated malabsorption, for example, iron, or vitamin B12. A range of symptoms and signs may be present, making it imperative for primary care physicians to recognize even subtle clues of malabsorption syndromes. Classic symptoms include diarrhoea, steatorrhea, weight loss, flatulence and post pandial pain. Non‐gastrointestinal manifestations may include unexplained altered liver function tests, anaemia of obscure origin, skin conditions, unexplained infertility, and osteoporosis [228, 229, 230, 231, 232, 233, 234].

### 4.2 Statement

In a primary care setting, nutritional status assessment should include patient history, physical examination (hand grip strength, anthropometric measurements), and point of care tests such as the concentration of serum albumin, blood cell count, and prothrombin time.

In primary healthcare, various scales and instruments are used to evaluate the nutritional status of patients, particularly in older individuals suspected of having sarcopenia [235, 236, 237]. Common assessments include body mass index, handgrip strength, and other tests aimed at capturing overall nutritional health. In the primary care setting, several laboratory tests are also used for identifying malabsorption issues, such as blood cell count, vitamins, ferritin, and albumin [238, 239]. Point of care testing has progressively increased in different subspecialities, including primary care [240], although these tests may not be available in all European countries. Point of care tests allow rapid evaluation, in a user friendly manner, of several important parameters, such as albumin, haemoglobin, and prothrombin time [241], which may be helpful in rasing the suspicion of malabsorption, thus decresing the time of referral to a specialist doctor.

### 4.3 Statement

Special groups, such as pregnancy, childhood or older age should alert the physicians to the symptoms and signs that could indicate a malabsorption syndrome and to the use the available point of care of other tests, so to refer the patient or treat accordingly.

Primary health care physicians are increasingly adept at recognizing malabsorption syndromes in children and identifying malnutrition and sarcopenia in the elderly [242, 243]. Pregnant women are typically referred to secondary care when malnutrition is suspected, in accordance with the guidelines of each country's national health service. Nevertheless, it appears that primary care physicians require additional support to adequately diagnose these syndromes. This could be achieved through the use of point of care tests (e.g., albumin, prothrombin time, haemoglobin), laboratory (e.g., complete blood count, coeliac serology, micro/macronutrients, protein electrophoresis, faecal elasatase) or functional tests (e.g., breath tests), combined with physical examinations, patient history, and symptom assessment [244].

### 4.4 Statement

**FIGURE 2:**
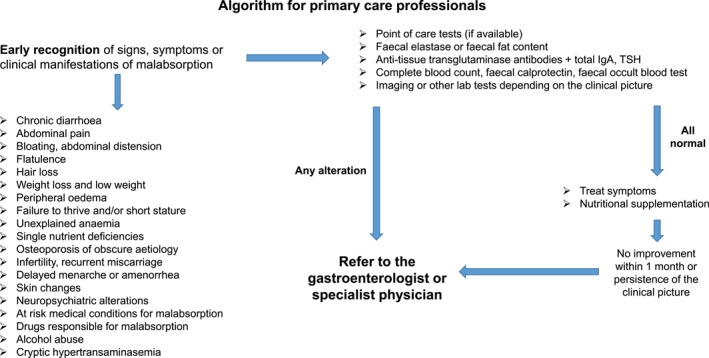
Schematic algorithm for the first assessment of a patient with malabsorption in a primary care setting. This algorithm should be considered as expert‐based, and the number and type of examinations may vary depending on specific clinical situations and local tests availability. In the primary care setting, the most important thing is to recognise signs and symptoms related to malabsorption as early as possible, as well as other differential diagnoses. According to the local test availability and to specific clinical situations, a series of first‐line tests should be performed. For example, among other laboratory tests, iron, vitamin B12, albumin, and folic acid may be useful as well in addressing the diagnosis, depending on the specific clinical setting. Coeliac disease is certainly one of the most common malabsorptive disorders, and therefore serology should always be performed. Faecal calprotectin is useful for differential diagnosis with inflammatory disorders of the colon. The availability of these tests may widely vary across different countries. The referral to a specialist physician shound be made within 1 month (or earlier if needed), depending on the clinical picture and the results of the first‐line tests, so to avoid treatment delay.

We propose a diagnostic algorithm for the initial assessment of patients with suspected malabsorption syndrome in a primary care setting (Figure 2).

In busy primary care settings, the use of tests depends upon the time required, their availability within each national healthcare system, and the permissions granted to primary health care physicians for ordering specific tests (e.g., there might be some restrictions in certain countries on requesting antibodies specific to CD). It is crucial that the primary healthcare settings are empowered to employ breath tests and simple blood or stool tests to conduct comprehensive assessments and prepare patients for specialist referrals [1, 233, 245]. Primary care physicians must be vigilant and capable of conducting straightforward diagnostic tests to rule out malabsorption syndrome when patients exhibit pertinent symptoms and signs. Should these tests return negative, malnutrition, along with malabsorption resulting from the use of certain medications like proton pump inhibitors or metformin should be considered. In cases where no improvement is observed, patients shall be referred for further evaluation by secondary and tertiary care.

### 4.5 Statement

Primary care physicians should follow the updated evidence‐based and consensus guidelines. Their specific role is:

- *To monitor and record the symptoms reported by the patients and inform specialised physicians or the treatment team (when present)*;
- *To assess*, *monitor and improve the nutritional status of the patients and inform the treatment team*;
- *To assess and improve the patient’ adherence to the treatment plan that has been provided by the specialists*;
- *To monitor*, *record and improve the psychological and mental health status of the patients*.

There is a general agreement that primary care physicians, in collaboration with specialists, can contribute to the treatment by providing special nutrients [225, 226] and offering psychological support to both patients and their families, contributing to the treatment's adherence. They should also monitor patients after any surgical procedures and report back to the specialists and treatment team [239, 242, 243, 246].

## 5 Conclusions

Malabsorption is a constantly evolving clinical challenge that involves several different medical specialities with gastroenterologists and internal medicine physicians usually being at the centre of the medical care. The diagnostic paradigm of malabsorption has markedly changed over the last centrury, especially due to the availability of disease‐specific tests, rather than tests looking for malabsorption. Nonetheless, early identyficantion of malabsorption as an entity still remains imperative in clinical practice, in order to avoid severe clinical consequences. In this first consensus on malabsorption, we have comprehensively covered the most important aspects of its diagnosis and management, by following strict rules for quality assurance. This consensus will hopefully be useful to practicing clinicians of different specialities, and we aim at updating it in the future.

## Author Contributions

M.V.L. and A.D.S. proposed the writing of the consensus to the UEG, they drafted the protocol, involved all scientific societies, and coordinated all the steps. All authors participated in all the steps, including literature review, first draft of the statements, voting, and writing of each statement. H.F.H., I.T., R.B., S.S., and A.F. acted as group leaders. M.V.L. wrote the final manuscript, collated and reviewed all statements, and critically reviewed the whole consensus. G.R.C. and A.D.S. critically reviewed the consensus and provided supervision through all the steps. All authors revised and approved the final version of the manuscript.

## Ethics Statement

The authors have nothing to report.

## Consent

The authors have nothing to report.

## Conflicts of Interest

The authors declare no conflicts of interest.

## Permission to Reproduce Material From Other Sources

The authors have nothing to report.

## Disclaimer

This consensus has been developed with reasonable care and with the best of knowledge available to the authors at the time of preparation. They are intended to assist healthcare professionals and allied healthcare professionals as an educational tool to provide information that may support them in providing care to patients. Patients or other community members using this consensus shall do so only after consultation with a health professional and shall not mistake this consensus as professional medical advice. This consensus must not substitute seeking professional medical and health advice from a health professional. This consensus may not apply to all situations and should be interpreted in the light of specific clinical situations and resource availability. It is up to every clinician to adapt this consensus to local regulations and to each patient's individual circumstances and needs. The information in this consensus shall not be relied upon as being complete, current or accurate, nor shall it be considered as inclusive of all proper treatments or methods of care or as a legal standard of care. UEG makes no warranty, express or implied, in respect of this consensus and cannot be held liable for any damages resulting from the application of this consensus, in particular for any loss or damage (whether direct or indirect) resulting from a treatment based on the guidance given herein. UEG shall not be held liable to the utmost extent permissible according to the applicable laws for any content available on such external websites, which can be accessed by using the links included herein.

## Data Availability

All data generated for developing this consensus have been included in the paper. No other data are available.
